# Editorial: Challenges and advances in global school health promotion

**DOI:** 10.3389/fpubh.2025.1575547

**Published:** 2025-03-10

**Authors:** Rafaela Rosário, Kevin Dadaczynski

**Affiliations:** ^1^School of Nursing, University of Minho, Braga, Portugal; ^2^Health Sciences Research Unit: Nursing (UICISA:E), School of Nursing of Coimbra, Coimbra, Portugal; ^3^Department of Health Sciences, Fulda University of Applied Sciences, Fulda, Hesse, Germany; ^4^Centre for Applied Health Sciences, Leuphana University Lueneburg, Lüneburg, Germany

**Keywords:** school, health promotion, complex intervention, wellbeing, whole school approach, Health Promoting School

Since the first International Conference on Health Promotion in 1986, settings have been recognized as fundamental to maintaining and promoting health. Despite terminological variations and conceptual advancements over the years, the core principle of the settings approach remains consistent, with an emphasis on the individual, social, and structural dimensions of health promotion (1). In its widely accepted definition, the World Health Organization (2) describes a setting as a “[…] place or social context in which people engage in daily activities in which environmental, organizational and personal factors interact to affect health and well-being”. Educational organizations such as kindergartens, schools, and universities serve as critical health-promoting settings, where social factors (e.g., climate, culture), organizational factors (e.g., leadership, management, professional development), and environmental factors (e.g., physical infrastructure, neighborhood context) all shape health and wellbeing outcomes. Moreover, these settings also provide sustained opportunities to develop health-related skills, attitudes, and behaviors from an early age that foster long-term wellbeing.

Shortly after the adoption of the Ottawa Charter, schools emerged as a key avenue for health promotion, largely because young people spend a significant portion of their waking hours in school (3) and can be easily reached there, regardless of their socioeconomic, cultural, or religious background. For instance, between 2000 and 2023, the number of pupils in primary and secondary schools worldwide has increased to approximately 1.41 billion (4). From a whole-school perspective, this also encompasses teachers, who exceeded more than 75.53 million worldwide in 2023 (4), along with school principals and non-teaching staff, all of whom represent a considerable portion of the global workforce. Against this background, extensive research has been conducted in recent decades, with the following emerging as the most common areas of focus: (i) mental health, wellbeing, and quality of life; (ii) health behaviors; (iii) oral health education; (iv) sexual and reproductive health education; and (v) principles and practices of health promotion in schools (5).

In terms of interventions, the landscape can be described as very heterogeneous, ranging from individual actions to curricular activities focusing on the individual knowledge and behaviors of pupils to complex Health Promoting School approaches addressing different target groups, topics and school levels. The multitude of developments has been brought together and continued at national and international levels, for example through networks (e.g., Schools for Health in Europe Network, UNESCO Chair Global Health & Education and its UNITWIN Network ‘Health and Education: Innovating for Sustainable Development'), conferences (e.g., European Conference on Health Promoting Schools) or compilations (6–8). Regularly collating and discussing the state of development is essential, particularly as new global challenges continue to emerge, requiring school health promotion to adapt and respond effectively. In addition to the long-term impact of the COVID-19 pandemic, these challenges encompass but are not limited to, escalating political conflicts and wars, the weakening of democracy, climate change, and rapid digital advancements (e.g., artificial intelligence). While these developments are driving progress in public health, they also present complex challenges to effectively navigating and managing health information (9, 10).

This editorial introduces the Research Topic, “Challenges and advances in global school health promotion,” which showcases a diverse collection of studies and research papers from around the globe. These address critical aspects of school health promotion and present innovative strategies and interventions aimed at creating healthy, more equitable educational environments. A total of 24 manuscripts were selected for publication after rigorous peer review.

The contributions of these 24 manuscripts, authored by 143 researchers and practitioners, exemplify a collective effort to advance understanding in this important area. These studies underscore a shared commitment to improving the health and wellbeing of children, families, and educational communities worldwide. By addressing pressing challenges and exploring innovative solutions, this body of research makes a valuable contribution to the implementation and refinement of effective school health promotion strategies.

## Addressing mental health and wellbeing

Several manuscripts in this Research Topic focused on the mental health and wellbeing of students and teachers, reflecting a growing recognition of the importance of psychological determinants of health in the school setting. For instance, the study by Granada-López et al. examined the mental health knowledge and classroom experiences of school teachers in Aragon, Spain, emphasizing the need for better training and resources to support students with mental disorders. Edwards et al. presented six evidence-based principles for whole-school wellbeing promotion, emphasizing the integration of wellbeing with key school goals, building on virtuous cycles, and evaluating wellbeing promotion by listening to different voices. Their research provides a comprehensive framework for schools to adopt and adapt existing practices to their own contexts. Finally, two articles focused on the perspective of school staff. Svensson and Warne explored the perspectives of school staff in Sweden managing student mental health issues and highlighted the need for more resources and greater organizational support. Seibt and Kreuzfeld investigated the relationship between working hours, mental health, and early retirement among part-time teachers in Germany. Their findings suggest that reducing teaching hours alone does not improve mental health, but that good mental health significantly increases the likelihood of regular retirement. This underscores the need for comprehensive mental health support for teachers.

## Promoting physical activity and healthy lifestyles

The promotion of physical activity and healthy lifestyles is a central theme in many articles in this Research Topic. The study by Domínguez-Martín et al. explored the relationship between self-perceived physical literacy and obesity-related outcomes among Spanish adolescents, highlighting the crucial role of physical literacy in maintaining a healthy weight and fitness levels. Additionally, the ACTIVE CLASS study by González-Pérez et al. investigated the effects of incorporating physical activity into academic classes, showing promising results in improving both health indicators, and educational outcomes. Using the theory of planned behavior, Jha et al. evaluated a school-based intervention regarding its potential to improve behavioral intentions to engage in regular physical activity among adolescents in India. Einhorn et al. analyzed changes in self-reported health and wellbeing indicators among primary school children in Wales from 2014 to 2022. Their study revealed a decline in physical health and unhealthy eating habits, and an increase in mental health issues, highlighting the urgent need for targeted interventions and policy focus to reverse these trends. The study by Murofushi et al. examined the multidimensional perspectives of underweight among emerging women in Japan, focusing on their dieting experiences. Given that 24% of the study population was found to be underweight, the research highlighted the need for tailored health education programs that address the specific needs of underweight women, whether they have dieted or not. Jiang et al. explored the challenges and countermeasures of research on sports for adolescent socialization in China. The authors' data mining study identified key themes and highlighted the need for better alignment between public demand and research focus to address the imbalance and inconsistency in current research efforts.

## Enhancing health literacy

The growing relevance of being able to use health-related information (health literacy) is also becoming increasingly important in the context of school health promotion. Rangnow et al. provided insights into the digital health literacy of primary and secondary school teachers in Germany, identifying key areas where teachers need support to use digital health information effectively. This is complemented by the work of Fiordelli et al., who developed a training program to enhance critical health literacy and scientific literacy among adolescents in Switzerland, demonstrating the feasibility and potential benefits of such interventions. Abdelkhalik et al. examined the effectiveness of a brief educational intervention in Lebanon to improve knowledge and attitudes regarding heart attack and cardiopulmonary resuscitation among high school students as part of a holistic school health promotion program.

## Addressing gender and socioeconomic disparities

Three articles explicitly address gender and socioeconomic disparities in school-based health promotion. Based on data from the HBSC study, Brindley et al. examined gender-specific social and environmental correlates of active travel to school in four European countries and found important differences in how boys and girls perceive and engage in active travel. The study by Horm et al. focused on optimizing health services for young children in poverty and argued for increased collaboration between Early Head Start programs and pediatric health care to better support vulnerable populations. Shenkman et al. explored the experiences of teachers in rural Western Kenya in building a supportive environment for menstrual health and hygiene and emphasized the role of school staff in addressing gender-specific health needs.

## Innovative interventions and methodologies

Innovative interventions and methodologies are showcased in multiple articles. For example, the study by Chhajer and Hira examined the impact of positive psychology and mindfulness-based interventions in natural settings on the wellbeing of Indian students, thus providing valuable insights for integrating nature into wellbeing programs. The Kairos study protocol by Gabaldón-Estevan et al. introduced a multidisciplinary approach to studying the effects of school schedules on health, wellbeing, and academic performance, highlighting the importance of aligning school schedules with students' biological rhythms. De Lorenzo et al. provided a mini review of the relationship between creativity and resilience in adolescents and young adults since the onset of the pandemic and suggested the need for more research in this area. Abrams et al. conducted a systematic review of sexual health promotion interventions in European schools, finding promising evidence for interventions that strengthen sexual health resources related to respect, communication skills, and attitudes. They recommended a comprehensive focus on multi-dimensional sexual health literacy in future research.

## Understanding implementation

Finally, the importance of policy and implementation is underscored by the contributions of McLoughlin et al. and Leksy et al.. In their study protocol, McLoughlin et al. described the development of measures that will allow practitioners and researchers to evaluate the implementation of school health policies through an equity lens. Based on the results of an initial screening of the preservice and in-service training for school principals, Leksy et al. emphasized the multiple perspectives of leadership in sustainable school health promotion and called for the systematic integration of health into professional development for school leaders. Bartelink et al. presented the results of a participatory research process where five key roles of school health advisors in the Netherlands were identified, providing insights into context-oriented support for schools.
